# Why indecisive trials matter: Improving the binocular rivalry imagery priming score for the assessment of aphantasia

**DOI:** 10.3758/s13428-025-02780-6

**Published:** 2025-08-04

**Authors:** Merlin Monzel, Christian O. Scholz, Joel Pearson, Martin Reuter

**Affiliations:** 1 Personality Psychology and Biological Psychology, Department of Psychology, University of Bonn, Kaiser-Karl-Ring 9, 53111 Bonn, Germany; 2 Institute of Philosophy II, Ruhr University Bochum, Bochum, Germany; 3 School of Psychology, University of New South Wales, Sydney, Australia

**Keywords:** Mental imagery, Binocular rivalry, Priming, Aphantasia, Diagnostics

## Abstract

Since mental imagery cannot be observed from the outside, it is all the more important to make it measurable. Yet, many so-called mental imagery tasks confuse object and spatial imagery or can be solved entirely without mental imagery, making them inappropriate for the assessment of mental imagery strength. One promising measurement method is the binocular rivalry task by Pearson et al. (Current Biology 18(13):982–986, 2008), which uses mental imagery priming to quantify mental imagery strength. Here, we propose an improved equation for the binocular rivalry priming score to significantly increase its predictive validity. In a sample of 38 aphantasics and 73 controls, we demonstrate that the binocular rivalry priming score calculated by the new equation explains more variance in the self-reported mental imagery capacity than the original equation. The improved binocular rivalry priming score is particularly beneficial when only a few trials are recorded (e.g., due to time or attention constraints) or when people with low mental imagery (i.e., aphantasics) have to be identified. The improved binocular rivalry priming score is advantageous in many situations, making it the preferred measure for future research.

**Supplementary Information:**

The online version contains supplementary material available at 10.3758/s13428-025-02780-6.

## Introduction

Visual imagery denotes the ability to create sensory representations of stimuli without corresponding external stimulation of the retinae (Pearson et al., 2015). As these representations are not directly observable from an outside perspective, it is important to make them measurable. This is especially true since the recent introduction of the term “aphantasia,” describing a neurocognitive phenomenon that is characterized by a marked reduction or absence of sensory mental imagery (Monzel et al., 2022; Zeman, 2024). To study the causes and consequences of aphantasia, it is necessary to develop tasks that can reliably and validly identify people with aphantasia and compare them with people who have imagery in or above the typical range (i.e., hyperphantasia; Zeman, 2024).

Aphantasia has recently become a topic of increased interest, partly due to findings showing that people with aphantasia can successfully perform tasks that were previously thought to rely on the use of (visual) mental imagery (e.g., Kay et al., 2024; for a discussion, see Scholz, 2024a). Furthermore, the condition does not impact the lives of the affected to the degree that it meets the criteria for a mental disorder (Monzel et al., 2023a, 2023b, 2023c), and many of the affected often report not even having been aware of their imagery deficit for decades of their lives (Scholz, 2024b; Zeman et al., 2020), thus questioning the importance commonly attributed to visualization for our overall cognitive functioning. However, it has been suggested that the observed competency of aphantasics might be explained by *unconscious* mental imagery (Nanay, 2021, 2023), meaning that, while aphantasics may not experience any mental imagery, they may nonetheless have the corresponding neuronal representation. Importantly, while there is indeed an ongoing debate about whether aphantasics utilize alternative strategies or unconscious imagery (e.g., Michel et al., 2025; Scholz et al., 2025), both camps agree that aphantasia most likely represents a heterogeneous category. However, to tell these hypothesized subgroups apart, next to needing reliable assessment tools, researchers have to apply tests that sidestep the experiential dimension and assess more indirect criteria associated with (unconscious) mental imagery.

In past research, several methods have been proposed to measure mental imagery. These methods can be divided into self-report questionnaires and indirect measures. Self-report questionnaires include the frequently used Vividness of Visual Imagery Questionnaire (VVIQ; Marks, 1973), the Plymouth Sensory Imagery Questionnaire (PSIQ; Andrade et al., 2014) and the Spontaneous Use of Imagery Scale (SUIS; Kosslyn et al., 1998). These types of tests assess qualitative aspects of the individual’s (subjective) experience, such as the vividness or detailedness of their imagery experience. However, as these measures are vulnerable to the limitations of introspection and memory, it has been suggested that such self-report questionnaires cannot be used to reliably assess one's mental imagery (Schwitzgebel, 2002). To overcome these limitations, various indirect measures have been proposed for assessing mental imagery, including behavioral tasks and physiological and neuroimaging measures (see Zeman, 2024). Several behavioral tasks were developed to measure mental imagery, such as mental rotation tasks (for a review, see D. G. Pearson et al., 2013). However, these tasks often confuse object imagery and spatial imagery (Blajenkova et al., 2006; Blazhenkova, 2016), although both can be dissociated in normal imagers (Burton, 2003; Burton & Fogarty, 2003) as well as aphantasics (Bainbridge et al., 2021). Visual detail questions are also frequently used to measure mental imagery, but can also be solved by aphantasics (Liu & Bartolomeo, 2023; Milton et al., 2021), probably due to alternative approaches such as self-embodied or analytic strategies (Kay et al., 2024; Monzel et al., 2024a, 2024b). Some physiological measures have also been proposed to measure mental imagery. For example, it was found that aphantasics, compared to controls, showed a lower or nonexistent galvanic skin response when reading scary stories (Wicken et al., 2021) and a reduced pupillary light reflex when attempting to imagine bright objects (Kay et al., 2022). However, such physiological measures require special equipment, and sensitivity and specificity coefficients are rather low (Monzel et al., 2024a, 2024b). Lastly, neuroimaging assessments are often too cost-intensive and not efficient enough to be widely used for the assessment of mental imagery (e.g., Cui et al., 2007).

This leads us to the binocular rivalry task by J. Pearson et al. (2008), a relatively new task whose mental imagery priming score was shown to be associated with self-reported mental imagery (J. Pearson et al., 2011; Wagner & Monzel, 2023). Moreover, the task was able to distinguish between aphantasics and controls in several studies (Kay et al., 2022; Keogh & Pearson, 2018, 2024; Monzel et al., 2024b). In contrast to previous mental imagery tasks, spatial imagery is not directly involved in the binocular rivalry task, as the participants must imagine a specific stimulus (color and orientation) and not its spatial relationship to other objects. There are even experiments that provide evidence that alternative strategies, such as attentional shifts, are not sufficient to increase the priming score (Keogh & Pearson, 2021; for a discussion, see Krempel & Monzel, 2024). The task has also been used to test imagery capacity (Keogh & Pearson, 2017), and predicts the pupil-difference scores using pupillometry to measure imagery (Kay et al., 2022), there are versions that are detection-based as well, using probes in the rivalry stimulus, which therefore do not require subjective reports of perceptual dominance in the binocular rivalry stimulus (Chang & Pearson, 2018). Furthermore, performance in the task also seems to be influenced by unconscious mental imagery (Kwok et al., 2019; Purkart et al., 2025), making it an indicator of imagery strength regardless of whether mental imagery is conscious or not. Ultimately, this allows us to distinguish between the hypothesized subgroups of aphantasics who genuinely lack mental imagery and those who may have unconscious imagery. Lastly, apart from a computer and software, the only equipment needed for a binocular rivalry task is anaglyph glasses, which are rather cheap, making it widely available. Thus, we propose that the binocular rivalry task is one of the most promising tasks to reliably and validly measure mental imagery strength.

**Fig. 1:**
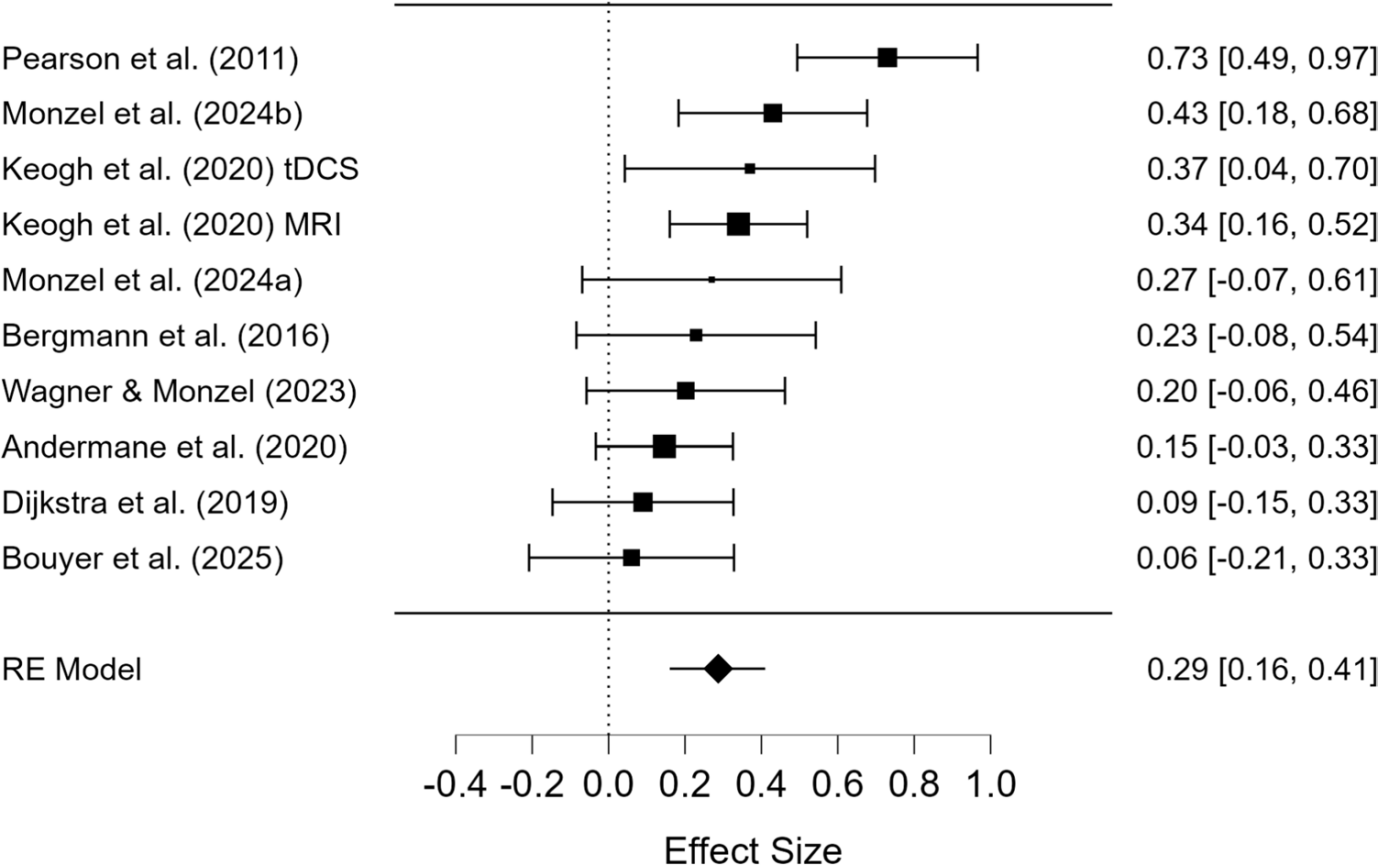
Forest plot for the correlations between the subjective dominance and self-reported mental imagery for the studies presented in Bouyer et al. (2025)

Admittedly, not all studies find a significant association between the binocular rivalry score and self-reported mental imagery vividness (e.g., Andermane et al., 2020; Bergmann et al., 2016), as discussed in Bouyer et al. (2025). However, when the data from all studies presented in Table 1 in Bouyer et al. (2025) are meta-analytically aggregated (DerSimonian-Laird method), the association between the binocular rivalry score and self-reported mental imagery is still significant, *r* = 0.29, *p* < 0.001, even when the most influential effect size from Pearson et al. (2011) is excluded (std. residual = 3.78), *r* = 0.23, *p* < 0.001, to achieve homogeneity, *Q*(8) = 8.48, *p* = 0.388,* I*^2^ = 5.6% (see Fig. 1).^1^ Importantly, Bouyer et al. (2025) are not arguing against visual imagery priming binocular rivalry dominance in general, but against a robust association between self-reported mental imagery and subjective binocular rivalry dominance. Thus, it is important to improve the binocular rivalry score to make the association more robust and applicable for measuring mental imagery strength.

**Table 1:**
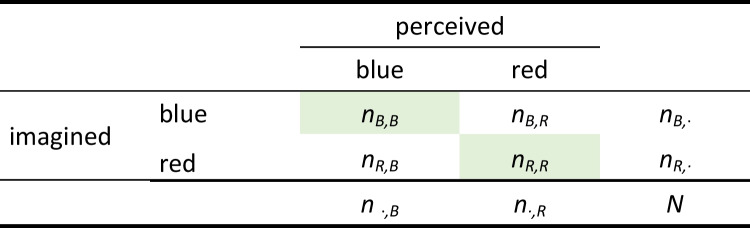
Cross table after the exclusion of mixed trials

The green highlighted values depict trials which were successfully primed, i.e., trials in which participants imagined red and perceived red (*n*_*R,R*_) and trials in which participants imagined blue and perceived blue (*n*_*B,B*_)

### The binocular rivalry task

In the binocular rivalry task, participants are presented with two differently colored Gabor patterns, each to one eye, for example, using anaglyph glasses. When participants are asked to imagine one of the two differently colored Gabor patterns beforehand, the perception of the rivalry becomes biased, leading to a more dominant perception of the previously imagined pattern. After each rivalry presentation, participants are asked to indicate which of the Gabor patterns they perceived: a horizontal-blue pattern, a vertical-red pattern, or a mixed pattern. A mixed response can be given in the case that an observer is “unable to distinguish which grating had appeared more dominant due to binocular combination or piecemeal rivalry” (Rademaker & Pearson, 2012).^2^ Before running the task, the rivalry stimulus is calibrated in a way that a priori eye dominance is eliminated, that is, neither stimulus should be more dominant. For the calculation of the binocular rivalry priming score, mixed answers are excluded. Typically, when the total percentage of mixed trials is above a given threshold—for example, 20%—participants are excluded completely (e.g., J. Pearson et al., 2008, 2011; Rademaker & Pearson, 2012). This leads to a cross table as depicted in Table 1.

The binocular rivalry priming score is subsequently calculated as the proportion of trials in which the same Gabor pattern was perceived as previously imagined (congruent trials), since the likelihood of seeing the previously imagined Gabor pattern increases with mental imagery strength:

$${\%}_{primed}=\frac{{n}_{B,B }+{n}_{R, R} }{{n}_{B, B }+{n}_{R, R}+ {n}_{B, R }+{n}_{R, B}}$$

### Improving the binocular rivalry priming score

An important shortcoming of the binocular rivalry task is that mixed trials are excluded from the calculation of the binocular rivalry priming score, which makes the paradigm either less efficient if additional non-mixed trials need to be recorded as substitutes, or less reliable if no additional non-mixed trials can be recorded due to time or attention constraints (for the mathematical association between trial number and reliability, see Cortina, 1993). Moreover, the answer option “mixed” cannot be excluded from the paradigm, as the random selection of one of the other two answer options would reduce the validity of the binocular rivalry priming score when the binocular rivalry display had actually been perceived as neither predominantly red nor blue. Following this, mixed trials contain some degree of information, i.e., that neither the congruent Gabor pattern nor the incongruent Gabor pattern was perceived dominantly, leading to an extended cross table as depicted in Table 2.

**Table 2:**
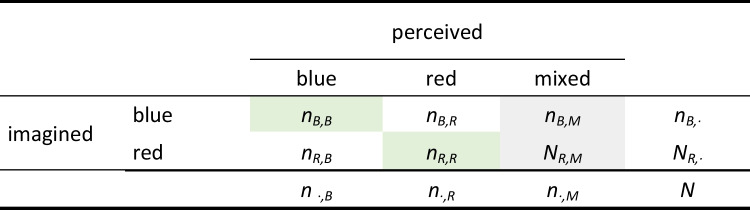
Cross table without the exclusion of mixed trials

The green highlighted values depict trials which were successfully primed, i.e., trials in which participants imagined red and perceived red (*n*_*R,R*_) and trials in which participants imagined blue and perceived blue (*n*_*B,B*_). Gray highlighted values indicate the number of mixed trials (*n*_*B,M*_, *n*_*B,M*_)

Mathematically, the significance of mixed trials can be expressed by halving their weighting in the numerator, whereas they are fully weighted in the denominator:

$${\%}_{primed}= \frac{{n}_{B, B }+{n}_{R, R}+0.5*{n}_{B, M }+0.5*{n}_{R, M} }{{n}_{B, B }+{n}_{R, R}+ {n}_{B, R }+{n}_{R, B}+ {n}_{B, M }+ {n}_{R, M}}$$

**Fig. 2:**
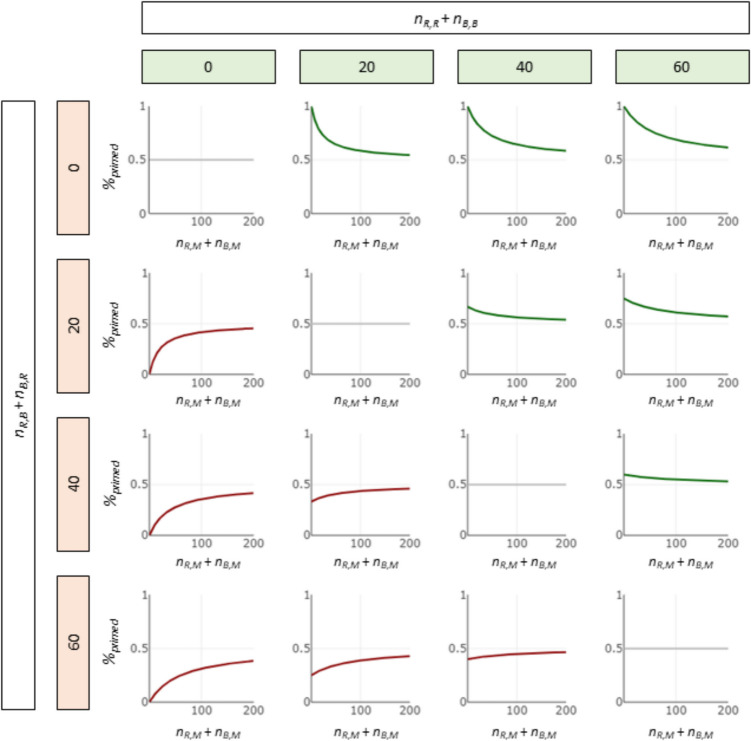
Line plot matrix to visualize the association between number of mixed trials (*x*-axes) and priming score (*y*-axes) for different numbers of congruent (columns 0, 20, 40, 60) and incongruent trials (rows 0, 20, 40, 60). When neither the congruent nor incongruent Gabor pattern becomes dominant, the mixed trials exert no influence (gray curves). The higher the proportion of congruent trials, the higher the position of the priming score (above 0.5, green curves). The higher the proportion of incongruent trials, the lower the position of the priming score (below 0.5, red curves). The higher the number of congruent and incongruent trials, the smaller the influence of the mixed trials (= flatter curves)

Due to the halved weighting of the mixed trials in the numerator, the term tends towards 0.5 the more mixed trials are measured, which indicates that there is no priming, since the calibrated baseline was observed (i.e., neither clearly red nor blue Gabor patterns). Importantly, mixed trials alone never lead the priming score to cross the 0.5 limit, as they favor neither congruent nor incongruent Gabor patterns. Instead, the proportion of congruent and incongruent Gabor patterns determines the general position of the priming score (i.e., below or above the 0.5 limit), and the total number of mixed trials determines the asymptotic convergence of the priming score to this position (see Fig. 2). Thus, the informative value of mixed trials can be preserved mathematically without being overinterpreted in relation to non-mixed trials.

### Advantages of the improved binocular rivalry priming score

Besides preserving the informative value of non-mixed trials and adding the informative value of mixed trials, the improved equation of the binocular rivalry priming score has some specific advantages in the assessment of aphantasia.^3^ As explained above, the reliability of diagnostic tasks depends on the number of repeated measurements (Cortina, 1993), which is limited by time or attention constraints, resulting in lower reliability when the original equation of the binocular rivalry priming score is used and a large number of mixed trials is recorded. In the case of aphantasia, however, a high number of mixed trials could be hypothesized, since aphantasics, at least those without unconscious mental imagery, cannot be primed by their mental images and should therefore not be biased to seeing either color more dominantly (Keogh & Pearson, 2018, 2024; Monzel et al., 2024b)—at least if one assumes that the visual memory from the prior rivalry presentation also vanishes in aphantasics and therefore does not stabilize perceptual dominance. In this case, using the improved equation, the number of repeated measurements is preserved, making the measurement more reliable. Alternatively, it can be hypothesized that aphantasics are primed by the color they perceived in the previous trial. In this case, we would not find an increased proportion of mixed trials in aphantasics, but increased perceptual stability (see J. Pearson et al., 2008).

Regardless of which outcome, the improved binocular rivalry priming score equation should be particularly advantageous when (a) time and attention constraints are too high to collect additional trials or (b) a high number of mixed trials is expected. Moreover, in this way, no participants have to be excluded due to a high proportion of mixed trials (e.g., as in J. Pearson et al., 2008). To demonstrate that these advantages are not only theoretical but also of practical value, we will examine the incremental validity of the improved binocular rivalry priming score in predicting our participants’ self-reported vividness of visual imagery using actual data.

## Method

### Dataset

**Fig. 3:**
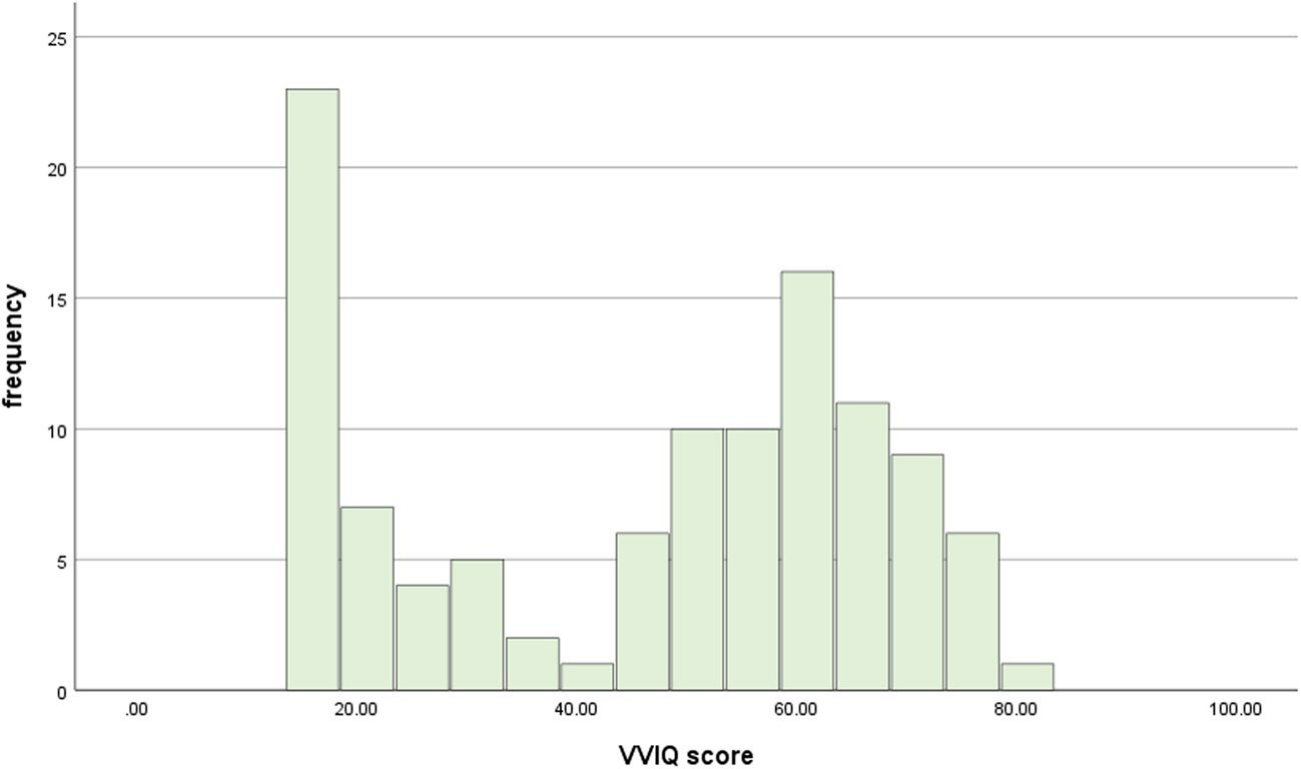
Distribution of VVIQ scores within our sample. The two peaks are characteristic for aphantasics and controls

We used the dataset by Monzel et al., (2023a, 2023b, 2023c), including 111 participants who completed the binocular rivalry task. The sample consisted of 38 people with aphantasia (VVIQ ≤ 32, criterion according to Dance et al., 2022) and 73 people without aphantasia. The distribution of the VVIQ is depicted in Fig. 3.

### Procedure and data cleaning

Before the start of the binocular rivalry task, an eye dominance calibration was implemented (see Supplemental Material). For each trial in the binocular rivalry task, participants were asked to imagine either a red-horizontal or a blue-vertical Gabor pattern, indicated by the presentation of a white letter cue (“R” vs. “B”) on a black screen for 750 ms. The cues were chosen pseudo-randomly to ensure the same number of each cue. This procedure was chosen to avoid systematic response patterns (e.g., alternating responses due to the presentation of cued-suppressed trials only), despite cue-dominant trials not being able to distinguish between effects of mental imagery priming and effects of perceptual stability, as both the previous mental image and the previous percept would be the same.^4^ The imagery period was 6,000 ms. Afterwards, participants were presented with the binocular rivalry stimulus on a black screen for 750 ms. A total of 12.5% of the trials presented mock rivalry displays, consisting of either only blue or only red Gabor patterns. The experiment was terminated as soon as 32 non-mock trials were completed (for two participants, 33 trials were collected due to a coding error). For the calculation of the priming score, we considered only those datasets in which a maximum of one mock trial was answered incorrectly, leading to no exclusions. Three people with one wrong answer in the mock trials remained in the dataset. Overall, there was no imagery-consistent bias on mock trials, *t*(110) = 0.30, *p* = 0.762.

### Statistical analyses

First, we visually inspected the distribution of both binocular rivalry priming scores to assess the impact of the improved equation. A correlation was calculated between the difference of the original and the improved binocular rivalry priming scores and the number of non-mixed trials to demonstrate that the two scores became increasingly similar as fewer mixed trials were recorded. Next, we used regression analyses to predict the VVIQ score of our participants with the original binocular rivalry priming score and the improved binocular rivalry priming score. Afterwards, a correlation comparison was conducted to test whether the improved priming score explained significantly more variance in subjective vividness reports than the original priming score. Next, we used regression analyses to predict the absolute prediction error between observed and predicted VVIQ scores, with the observed VVIQ scores demonstrating that the improved priming score minimized the absolute prediction error, especially for people with low mental imagery (aphantasia). Lastly, we examined the influence of the total number of non-mixed trials. For this, we used a median split, separating the dataset into participants with a low and a high number of non-mixed trials. We then repeated the regression analysis with the binocular rivalry priming scores as the predictor and the VVIQ scores as the criterion in each of the two subsets. Correlation comparisons in each subset were used to check whether one of the two equations surpassed the other. All analyses were performed with JASP (JASP Team, 2024). Bayes statistics were calculated using the default priors of JASP.

## Results

### The integration of mixed trials

**Fig. 4:**
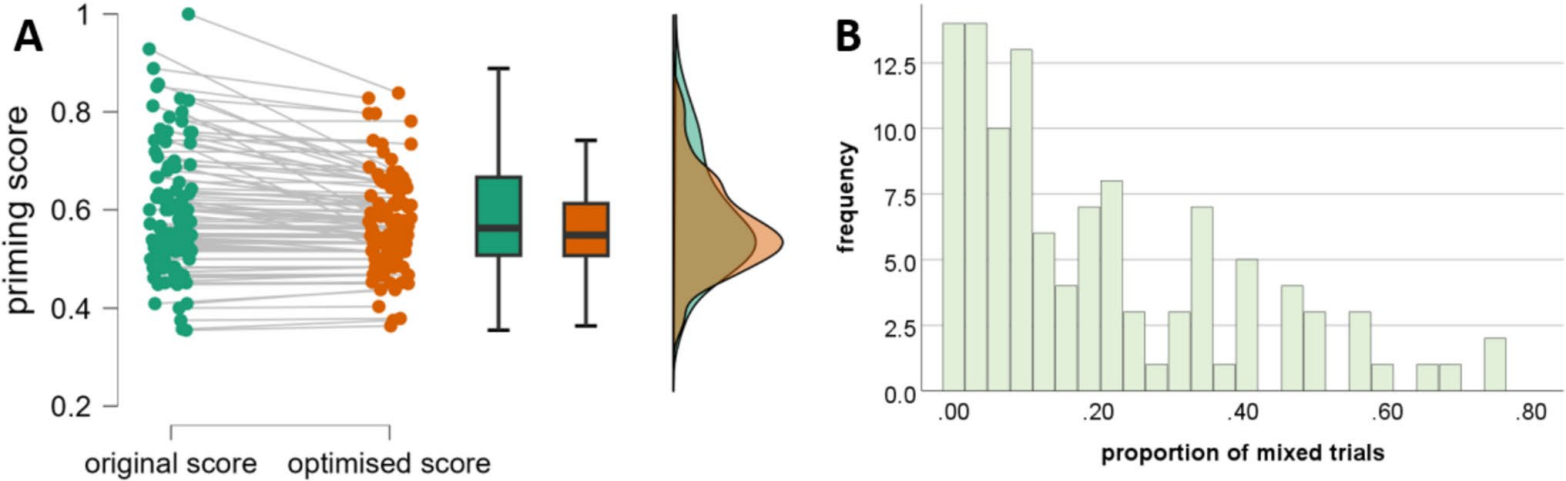
**A** Individual pairs of the binocular rivalry priming score according to the original or improved equation. The distribution of the improved binocular rivalry priming score is slightly narrower than the distribution of the original binocular rivalry priming score. **B** Distribution of the proportion of mixed trials. A high proportion of mixed trials led to stronger attenuation of the original binocular rivalry priming score than a lower proportion of mixed trials

The distributions of the binocular rivalry priming scores according to the original and improved equations are depicted in Fig. 4a. As expected, for most participants, the two binocular rivalry priming scores were nearly the same. However, some extreme values regressed to the mean, most likely due to a less reliable measurement of the original binocular rivalry priming score caused by a high number of mixed trials (for a distribution, see Fig. 4b). This assumption was confirmed by a significant association between the number of non-mixed trials and the absolute difference between the two binocular rivalry priming scores, *r*(109) =  − 0.73, *p* < 0.001, *BF*_10_ = 2.92 × 10^16^, indicating that a lower number of mixed trials in the original equation led to more similar binocular rivalry priming scores. While this effect can be reduced by excluding participants with a high proportion of mixed trials (e.g., > 20%), the effect is still clearly present, *r*(66) =  − 0.46, *p* < 0.001, *BF*_10_ = 308.21.

**Fig. 5:**
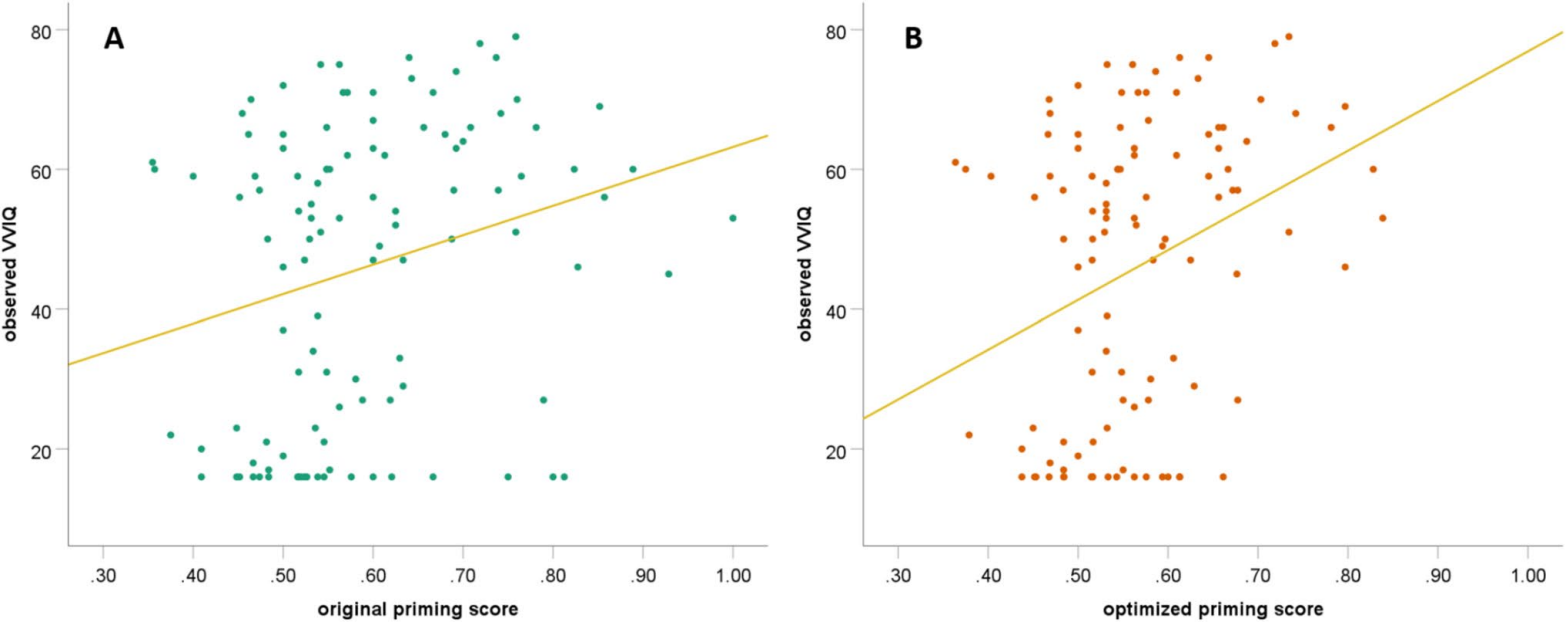
The original priming score (**A**) and the improved binocular rivalry priming score (**B**) predicting the VVIQ score

Next, the association between both binocular rivalry priming scores and the VVIQ was examined. The VVIQ scores were significantly predicted by the binocular rivalry priming score using the original priming equation, β = 0.253, *p* = 0.007, *BF*_10_ = 5.42, *R*^2^ = 0.056, as well as by the new binocular rivalry priming equation, β = 0.319, *p* < 0.001, *BF*_10_ = 42.50, *R*^2^ = 0.093 (see Fig. 5). However, the improved binocular rivalry priming score explained approximately 1.66 times more variance in the VVIQ data than the original binocular rivalry priming score, *z* = 2.53, *p* = 0.006, indicating an improvement in the equation, which is also reflected in the strong increase in the Bayes statistic, where values above 5 indicate substantial evidence for H1 compared to H0, and values above 30 indicate very strong evidence (Lee & Wagenmakers, 2013).

Lastly, we checked the influence of the number of non-mixed trials by separating the dataset into participants with a low and a high number of non-mixed trials using a median split: While the incremental validity of the improved binocular rivalry priming score was given when a low number of non-mixed trials was recorded (*Mdn* = 21, *r*_opt_ = 0.32, *p* = 0.017, *r*_orig_ = 0.22, *p* = 0.117), *z* = 2.12, *p* = 0.017, the improved binocular rivalry priming score did not explain more variance when a high number of non-mixed trials was recorded (*Mdn* = 30, *r*_opt_ = 0.31, *p* = 0.019, *r*_orig_ = 0.31, *p* = 0.021), *z* = 0.86, *p* = 0.194. Thus, the informational value of mixed trials decreases the more non-mixed trials can be recorded.

### The importance of perceptual stability in aphantasics

Aphantasics (*M* = 0.22, *SD* = 0.22) did not show a significantly higher proportion of mixed trials than controls (*M* = 0.19, *SD* = 0.17), *t*(62.63) = 0.65, *p* = 0.520, *g* = 0.14, *BF*_10_ = 3.94. Following this, we checked whether this was because of perceptual stability in aphantasics. Since many trials of the binocular rivalry task are presented in a row, it is possible that the dominant rivalry pattern from the previous trial may prime the next trial. Since this process is not interrupted by mental imagery in aphantasics, they likely show perceptual stability instead of mental imagery priming (see Pearson et al., 2008). Indeed, perceptual stability was higher for aphantasics (*M* = 0.83, *SD* = 0.13) than for controls (*M* = 0.76, *SD* = 0.15), *t*(109) = 2.69, *p* = 0.008, *g* = 0.53, *BF*_10_ = 4.92.

**Fig. 6:**
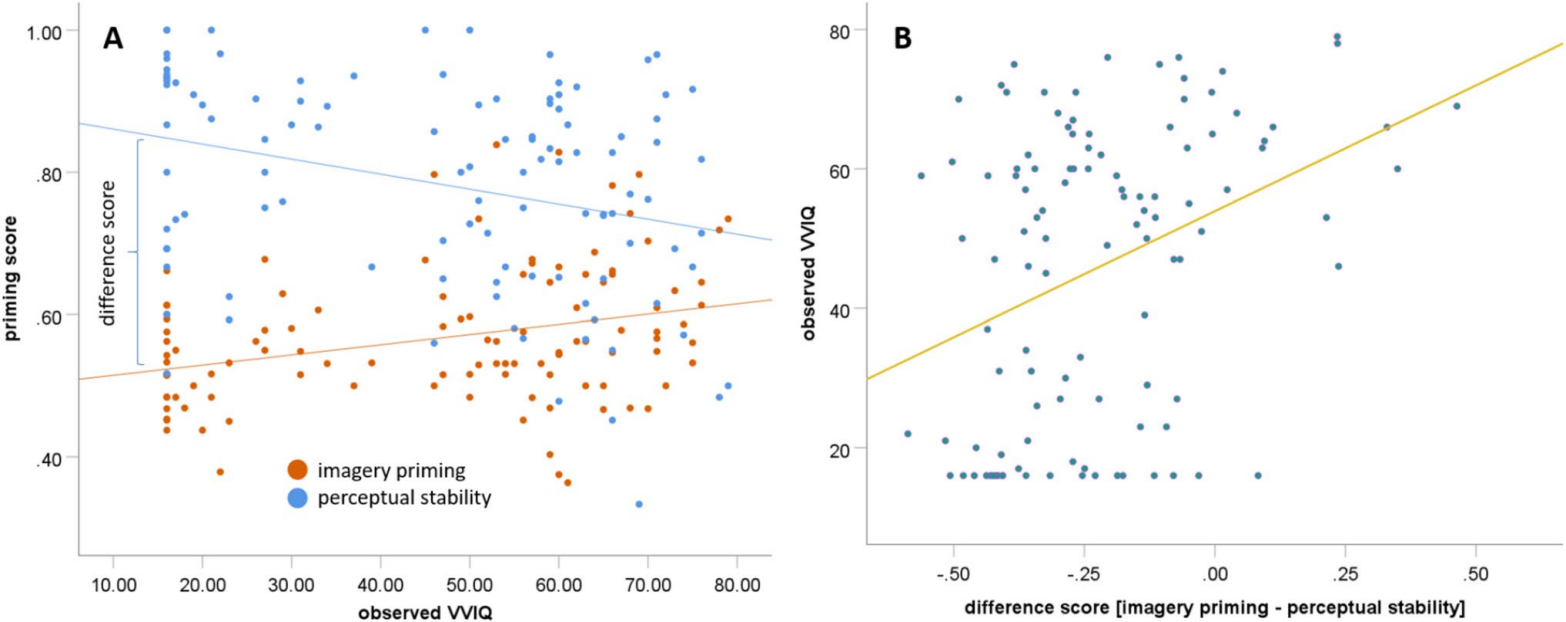
The observed VVIQ scores predicting the mental imagery and perceptual stability (**A**) and their difference score predicting the VVIQ (**B**)

Next, we examined how this information could be integrated into the binocular rivalry score. As can be seen in Fig. 6a, the regression between VVIQ and improved mental imagery priming, β = 0.319, *p* < 0.001, *BF*_10_ = 42.50, *R*^2^ = 0.093, and VVIQ and perceptual stability, β =  − 0.305, *p* < 0.001, *BF*_01_ = 25.86, *R*^2^ = 0.093, was in opposite directions. Thus, while an increase in mental imagery leads to more mental imagery priming, it also leads to a decrease in perceptual stability, since the mental image between the binocular rivalry stimuli disrupts the neural representation of the previously dominant color. Knowing this, a difference score between the perceptual stability score and improved mental imagery priming score can be calculated, which is largest for aphantasics and smallest for hyperphantasics. Using this difference score, the predictive validity for the VVIQ can be further improved, β = 0.358, *p* < 0.001, *BF*_10_ = 192.38, *R*^2^ = 0.128 (see Fig. 6b).

### Categorical differences between aphantasics and controls

As can be seen in Fig. 2, the distribution of VVIQ ratings was clearly bimodal in the present sample due to targeted data collection from aphantasics. While regression analysis is robust even for bimodal distributions (Zeller & Levine, 1974), regression analyses within these two groups can reveal whether the effect was driven by these two extremes or by the whole mental imagery spectrum. Thus, we reran the analysis with the most predictive binocular rivalry score, that is, the binocular rivalry difference score, separately for aphantasics and controls. Within aphantasics, no significant association between VVIQ and binocular rivalry difference score was found, β = 0.078, *p* = 0.640, *BF*_01_ = 2.91, *R*^2^ = 0.006, whereas in controls, the association between VVIQ and binocular rivalry difference score just missed significance, β = 0.227, *p* = 0.053, *BF*_10_ = 1.24, *R*^2^ = 0.038. Thus, the binocular rivalry task is highly suitable for distinguishing between aphantasics and non-aphantasics, but less suitable for differentiating within the groups. Importantly, however, this could also be driven by variance restrictions within the groups, especially since the regression within the higher variance subgroup, that is, the control group, was almost significant.

## Discussion

As shown in our analyses, the improved binocular rivalry priming score explains more variance in our participants’ vividness of visual imagery than the original binocular rivalry priming score when relatively few non-mixed trials are recorded or when the proportion of mixed trials is high. However, since the improved binocular rivalry priming score becomes increasingly similar to the original binocular rivalry priming score with fewer mixed trials, the improved binocular rivalry priming score is never worse than the original score, even when a low proportion of mixed trials and a high number of non-mixed trials are recorded. Also, a small number of mixed trials does not influence the improved binocular rivalry priming score, as the influence of mixed trials becomes less significant for participants with many non-mixed trials (see Fig. 2). Thus, the improved binocular rivalry priming score is more favorable. In fact, when no mixed trials are recorded, the two equations become equivalent:

$${\%}_{primed}\left({n}_{B, M }=0, {n}_{R, M}= 0\right)= \frac{{n}_{B, B }+{n}_{R, R}+0.5*0+0.5*0 }{{n}_{B, B }+{n}_{R, R}+ {n}_{B, R }+{n}_{R, B}+ 0+ 0}=\frac{{n}_{B,B }+{n}_{R, R} }{{n}_{B, B }+{n}_{R, R}+ {n}_{B, R }+{n}_{R, B}}$$

Aphantasics did not show more mixed percepts in the binocular rivalry task than controls. This could be explained by perceptual stability between two consecutive trials in aphantasics, whereas perceptual stability in controls was more likely to be disrupted by their own mental imagery (Pearson et al., 2008). Consequentially, perceptual stability, as the inverse of mental imagery priming, can also be included in a binocular rivalry difference score, showing further improvements in predicting mental imagery vividness (see Pearson et al., 2008).

### Practical implications

Since real-world applications of the binocular rivalry task are always affected by time and attention constraints, it is important to note that testing using the binocular rivalry task cannot be extended indefinitely. Thus, excluding mixed trials (or even participants due to mixed trial thresholds) is inefficient. Moreover, mental imagery is typically a voluntary task (but see Pearson et al., 2015) that requires compliance, and compliance declines over time. Thus, we want our measure to be as efficient as possible. Using the improved binocular rivalry priming score, the informative value of mixed trials can be preserved while simultaneously increasing the reliability of our measure due to the preserved number of repeated measurements. In addition, the use of mixed trials is advisable even from an ethical point of view, as the cost–benefit ratio for participants should always be optimized, and excluding trials with informative value would reduce the benefit while simultaneously increasing the cost, as participants would have to invest more time to collect more trials.

Importantly, the binocular rivalry score is especially useful in distinguishing between aphantasics and non-aphantasics, whereas its predictive validity shrinks within the groups. This could also explain why some of the previous studies did not find an association between mental imagery vividness and binocular rivalry bias, as, for example, Bouyer et al. (2025) only included participants with VVIQ2 values above 80 (roughly corresponding to 40 points in the VVIQ), omitting aphantasics completely.^5^ Considering this, a future practical application of the binocular rivalry task could be to validate extreme imagery groups, as has been done previously (e.g., Keogh & Pearson, 2018, 2024; Monzel et al., 2024a; Monzel et al., 2024b). It is important to note, however, that the observed correlation (β = 0.319) is still too small for a single case allocation. Nevertheless, since the binocular rivalry task is also able to measure unconscious imagery priming (Kwok et al., 2019), an additional application of the binocular rivalry task could provide incremental validity in distinguishing between the hypothesized subgroups of aphantasics who genuinely lack mental imagery and those who may have unconscious imagery—a differentiation that cannot be made using self-report questionnaires—given that these subgroups exist. Of note, perceptual stability between consecutive trials is less disrupted by mental imagery in aphantasics, which can also be incorporated in group validation, either separately or by using a difference score.

### Limitations

**Fig. 7:**
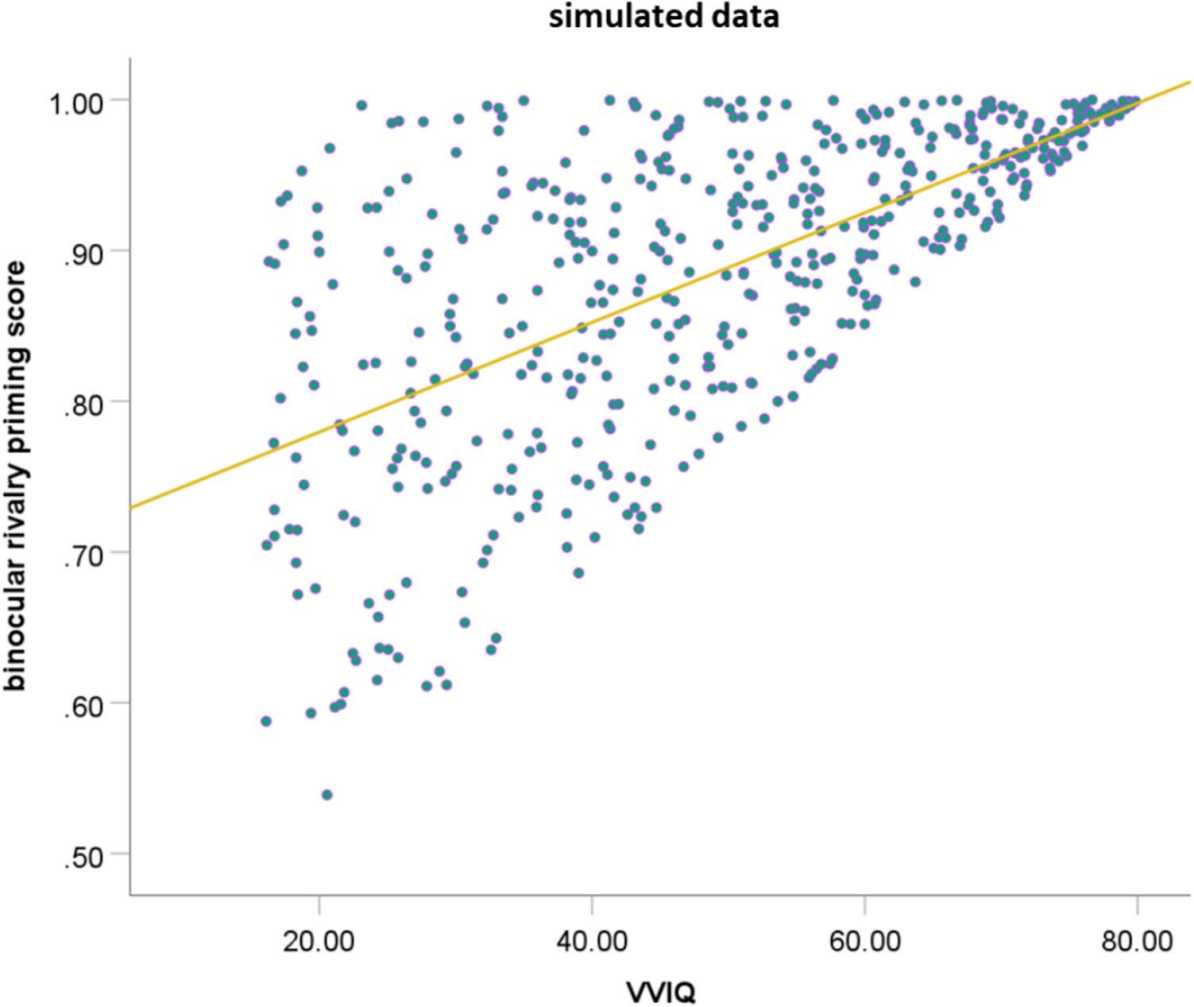
Simulated data assuming the existence of mental imagery representations without mental imagery experiences (= unconscious mental imagery) but denying the existence of mental imagery experiences without mental imagery representations. Association between self-reported mental imagery (VVIQ) and binocular rivalry priming score. While there might be people with unconscious mental imagery (VVIQ < binocular rivalry priming score), mental imagery experiences without mental imagery priming (VVIQ > binocular rivalry priming score) are rather implausible. Overall, this leads to a positive association between the VVIQ and binocular rivalry priming score (trend line)

The most obvious limitation in validating behavioral measures with self-report measures is that a behavioral measure validated with a self-report measure can never be better than the self-report measure itself. Is it thus possible that the binocular rivalry task identifies aphantasic skills (e.g., unconscious mental imagery) that are not captured by the VVIQ? For this, we have to remember that there are possibly two types of aphantasics: those who have unconscious mental imagery and those who have no mental imagery at all. Those with unconscious mental imagery would show high binocular rivalry scores but low VVIQ scores, while those with no imagery at all would show low binocular rivalry scores and low VVIQ scores. However, it is implausible, without a misinterpretation of instructions, that a person would report mental imagery in the VVIQ without underlying mental imagery representations that would be captured with the binocular rivalry task.^6^ This theoretical association can be simulated, generating only data points in which the performance in the binocular rivalry task is higher than or equal to the corresponding performance in the VVIQ (*n* = 1,000, see Fig. 7). For the simulation code, see Supplemental Material.

As can be seen from the regression line in Fig. 7, this configuration still leads to a positive correlation between VVIQ and the binocular rivalry scores, whose maximum value is *r* = 0.50. In fact, this makes the correlations of up to 0.36 that were found even more impressive. However, conceptually, when using the binocular rivalry score instead of/in addition to the VVIQ to identify aphantasics, the binocular rivalry score will only identify those without unconscious mental imagery, which would be in keeping with the VVIQ. Thus, despite being validated with a self-report questionnaire, it is conceivable that the binocular rivalry score has additional value compared to self-reports.

Lastly, it must be determined whether the response bias in the binocular rivalry task is actually driven by a perceptual influence (= *objective* mental imagery priming) or, alternatively, by participants being more willing to report seeing a pre-imagined input in ambiguous situations. Since mock trials are not ambiguous, an absence of bias in mock trials cannot exclude the latter possibility in rivalrous trials (see Bouyer et al., 2025, for a more extensive discussion).^7^ However, for our application, it seems inconsequential which mechanism leads to the binocular rivalry priming score, as long as the priming score is more predictive of mental imagery. Through the inclusion of mixed percepts, the data become more nuanced, be it because of partial priming or because participants’ selection of mixed percepts reflects more nuanced information when referencing their experiences against multiple subjective criteria (e.g., mixing ratio or stability of the percepts). Nevertheless, it can be asked which mechanism is more likely to be influenced by the inclusion of mixed trials. With regard to this point, we would argue that mixed trials can indicate a change from a stable percept to a mixed percept due to mental imagery priming, but also due to a greater willingness to include the pre-imagined input in the response; thus, our design is not made to disambiguate these mechanisms. However, we would argue that perceptual manipulations (e.g., bright luminance, Sherwood & Pearson, 2010) modulate the priming effect, suggesting that the effect itself is perceptual in nature. Of note, reported perceptual dominance and objective mental imagery priming are highly related (Bouyer et al., 2025).

## Conclusion

We were able to show that the improved equation for the mental imagery priming score of the binocular rivalry task by Pearson et al. (2008) is favorable in many situations, but never worse than the original equation. Thus, we propose the use of the new equation in future studies assessing mental imagery strength, especially in the assessment of aphantasia, since in aphantasia, mental imagery priming is less likely to occur. Overall, the binocular rivalry task is currently the most promising approach for assessing mental imagery strength without confounding it with other abilities, such as spatial imagery or analytic strategies.

## Supplementary Information

Below is the link to the electronic supplementary material.

Supplementary file1 (PDF 65.6 KB)

## Data Availability

The data and materials for all analyses are available at https://osf.io/9f8qu/?view_only=a4fef560c709492c9e60b3c4f3b133b7.

## Footnotes

1 The more conservative estimate from Bouyer et al. (2025) was used for the effect size measure of Wagner and Monzel (2023).

2 Although mixed percepts seem to be rather rare in the general population (e.g., 6.49% of rivalry trials; Rademaker & Pearson, 2012), Bouyer and Arnold (2024) report an aphantasic who experienced exclusively piecemeal rivalry. However, it is important to note that this was a single case study.

3 In this paper, we avoid the term “diagnosing aphantasia,” as we do not consider aphantasia to be a disorder (Monzel, Dance, et al., 2023a, 2023b, 2023c; Monzel, Vetterlein, et al., 2023a, 2023b, 2023c).

4 Given that perceptual stability also occurs in aphantasics, cue-dominant trials should not have an influence on the binocular rivalry score. Statistically, this is shown in a significant intercorrelation between the overall binocular rivalry priming score and a binocular rivalry priming score in cue-suppressed trials, *r*(109) =.77, *p* <.001, but not between the overall binocular rivalry priming score and a binocular rivalry priming score in cue-dominant trials, *r*(109) =.14, *p* <.137.

5 Importantly, Bouyer et al. (2025) found differences within participants, comparing high versus low imagery trials. These fluctuations in mental imagery vividness are neglected in the case of averaged mental imagery ratings. However, in aphantasics, no fluctuations are expected, leading to more stable measurements.

6 We take a physicalist viewpoint here and argue that a mental event cannot exist without a corresponding physical event in the brain, as is the prevailing view in neuroscience. However, opponents of physicalism may disagree.

7 Interestingly, many of our participants were surprised to learn that mock trials also existed between the rivalrous trials. However, we do not have systematic data on this point.

## Code availability

The code for all analyses is available at https://osf.io/9f8qu/?view_only=a4fef560c709492c9e60b3c4f3b133b7.

## Declarations

### Open practices statement

The data and code for all analyses are available at https://osf.io/9f8qu/?view_only=a4fef560c709492c9e60b3c4f3b133b7.
