# Supplementary material for: Why indecisive trials matter: Improving the binocular rivalry imagery priming score for the assessment of aphantasia
Source: Behav Res Methods. 2025 Aug 4;57(9):248. doi: 10.3758/s13428-025-02780-6 (PMC12321684; doi:10.3758/s13428-025-02780-6)
Supplement: Supplementary file 1 — Supplementary file1 (PDF 65.6 KB) [file 13428_2025_2780_MOESM1_ESM.pdf]

Supplemental Material

Eye dominance calibration

The eye dominance calibration procedure was a reprogramming of the procedure described in Pearson et al (2008). The aim of the procedure was to adjust the relative contrast of two gratings to determine the point at which perceptual competition was most balanced and thus most susceptible to interference. It has been shown that adaptation to a high-contrast stimulus leads to weaker neural responses the next time this pattern is presented during rivalry, increasing the likelihood of its perceptual suppression and reversal of perceptual dominance [1].

First, participants were shown a red-horizontal grating in full contrast for 7 seconds. Afterwards, they were presented with the rivalry display for 750 ms and asked to report which pattern they experienced more dominantly, selecting between the options 'red-horizontal', 'perfectly mixed', and 'blue-vertical'. The interstimulus interval was 3 seconds. In the next trial, participants were either presented with the red-horizontal or blue-vertical pattern, depending on which one appeared dominant in the previous rivalry display. This procedure was repeated for at least 10 non-mixed trials and then until a perceptual switch was induced in more than 80 % of non-mixed trials. Each time no perceptual switch was achieved, the contrast of the non-experienced grating was increased by 10 %.

1. Pearson, J., and Clifford, C.W. (2005). Mechanisms selectively engaged in rivalry: normal vision habituates, rivalrous vision primes. Vision Res. 45, 707–714.

For Review Only

Data simulation

To simulate data where the performance in the binocular rivalry task is greater than or equal to the performance in the VVIQ, only data points above the following line can be generated:

y_min = 0.0078 * x + 0.375

Using this function, a VVIQ value of x = 16 leads to a binocular rivalry priming score of y_min = .50, and a VVIQ value of x = 80 leads to binocular rivalry priming score y_min = 1.

1000 data points were simulated, where 1000 random x-values between 16 and 80 were drawn and assigned to random y-values between y_min and 1. The following SPSS code was used:

```
INPUT PROGRAM.
LOOP #i = 1 TO 1000.
   COMPUTE x = UNIFORM(64) + 16.
   COMPUTE y_min = 0.0078 * x + 0.375.
   COMPUTE y = UNIFORM(1.0 - y_min) + y_min.
   END CASE.
END LOOP.
END FILE.
END INPUT PROGRAM.
DATASET NAME SimulatedData.
EXECUTE.
```

For Review Only
